# Diagnostic Performance of Computed Tomography–Based Artificial Intelligence for Early Recurrence of Cholangiocarcinoma: Systematic Review and Meta-Analysis

**DOI:** 10.2196/78306

**Published:** 2025-09-18

**Authors:** Jie Chen, Jianxin Xi, Tianyu Chen, Lulu Yang, Kaijia Liu, Xiaobo Ding

**Affiliations:** 1 Department of Radiology The First Hospital of Jilin University Jilin China; 2 General Surgery Center Department of Hepatobiliary and Pancreatic Surgery The First Hospital of Jilin University Changchun China; 3 Department of Urology The First Hospital of Jilin University Changchun China

**Keywords:** artificial intelligence, computed tomography, cholangiocarcinoma, early recurrence, meta-analysis

## Abstract

**Background:**

Despite artificial intelligence (AI) models demonstrating high predictive accuracy for early cholangiocarcinoma recurrence, their clinical application faces challenges, such as reproducibility, generalizability, hidden biases, and uncertain performance across diverse datasets and populations, raising concerns about their practical applicability.

**Objective:**

This meta-analysis aims to systematically assess the diagnostic performance of AI models using computed tomography (CT) imaging to predict early recurrence of cholangiocarcinoma.

**Methods:**

A systematic search was conducted in PubMed, Embase, and Web of Science for studies published up to May 2025. Studies were selected based on the Participants, Index test, Target condition, Reference standard, Outcomes, and Setting (PITROS) framework. Participants included patients diagnosed with cholangiocarcinoma (including intrahepatic and extrahepatic locations). The index test was AI techniques applied to CT imaging for early recurrence prediction (defined as within 1 year), while the target condition was early recurrence of cholangiocarcinoma (positive group: recurrence; negative group: no recurrence). The reference standard was pathological diagnosis or imaging follow-up confirming recurrence. Outcomes included sensitivity, specificity, diagnostic odds ratio (DOR), and area under the receiver operating characteristic curve (AUC), assessed in both internal and external validation cohorts. The setting comprised retrospective or prospective studies using hospital datasets. Methodological quality was assessed using an optimized version of the revised Quality Assessment of Diagnostic Accuracy Studies-2 tool. Heterogeneity was assessed using the *I*² statistic. Pooled sensitivity, specificity, DOR, and AUC were calculated using a bivariate random-effects model.

**Results:**

A total of 9 studies with 30 datasets involving 1537 patients were included. In internal validation cohorts, CT-based AI models showed a pooled sensitivity of 0.87 (95% CI 0.81-0.92), specificity of 0.85 (95% CI 0.79-0.89), DOR of 37.71 (95% CI 18.35-77.51), and AUC of 0.93 (95% CI 0.90-0.94). In external validation cohorts, pooled sensitivity was 0.87 (95% CI 0.81-0.91), specificity was 0.82 (95% CI 0.77-0.86), DOR was 30.81 (95% CI 18.79-50.52), and AUC was 0.85 (95% CI 0.82-0.88). The AUC was significantly lower in external validation cohorts compared to internal validation cohorts (*P*<.001).

**Conclusions:**

Our results show that CT-based AI models predict early cholangiocarcinoma recurrence with high performance in internal validation sets and moderate performance in external validation sets. However, the high heterogeneity observed may impact the robustness of these results. Future research should focus on prospective studies and establishing standardized gold standards to further validate the clinical applicability and generalizability of AI models.

## Introduction

Among primary hepatic malignancies, cholangiocarcinoma is the second most lethal tumor, following hepatocellular carcinoma in biological aggressiveness [1]. Cholangiocarcinoma can be categorized into 2 primary subtypes—intrahepatic cholangiocarcinoma (IHC) and extrahepatic cholangiocarcinoma (EHC)—based on the specific anatomical location of biliary tract involvement [2]. Globally, the incidence of cholangiocarcinoma has been steadily increasing. Radical surgical resection remains the only recognized curative treatment; however, the long-term prognosis remains poor, with 5-year survival rates limited to 20%-35% [3]. More distressingly, the early recurrence rate (defined as recurrence within 1 year) following radical resection reaches as high as 40%-65% [4]. Li et al [5] identified early recurrence as an adverse prognostic factor after curative resection of cholangiocarcinoma. Accordingly, accurately predicting early recurrence in patients with cholangiocarcinoma after surgery has become an important clinical focus. Precisely identifying high-risk populations for early recurrence is of critical importance for guiding treatment decisions, developing individualized therapeutic strategies, and ultimately improving the prognosis of patients with cholangiocarcinoma [6,7].

The conventional diagnostic approaches for detecting cholangiocarcinoma recurrence include serum tumor biomarkers (notably CA19-9), imaging techniques such as computed tomography (CT), magnetic resonance imaging (MRI), ultrasonography, and pathological biopsy [8,9]. The diagnostic utility of serum biomarkers is compromised by their limited sensitivity and specificity, with performance substantially impacted by variables, including bilirubin concentrations and inflammatory states [10,11]. While traditional imaging modalities offer noninvasive insights into the tumor microenvironment, they are fundamentally constrained by inherent methodological limitations [12]. These approaches mainly rely on morphological features and basic quantitative measurements, which are often subjective and susceptible to interobserver variability [13]. As a result, they may not fully reflect the complex biological features of the tumor beyond what is visually apparent, reducing diagnostic accuracy. Pathological biopsy, on the other hand, has several challenges. It is invasive and prone to sampling bias, and it does not fully reflect the complex heterogeneity of the tumor. Moreover, its main use is for postsurgical assessment, which greatly limits its value for preoperative risk evaluation and stratification [14,15].

Recent studies have demonstrated significant predictive performance of artificial intelligence (AI) models, with some reports showing area under the receiver operating characteristic curve (AUC) values approaching 0.99 for predicting early recurrence in cholangiocarcinoma [16,17]. However, the clinical application of these technologies remains controversial. The field faces critical challenges, including model reproducibility, generalizability, and maintaining consistent performance across different patient populations and imaging protocols. There is also uncertainty about model performance on independent datasets, largely due to hidden biases and the lack of extensive external validation [14,18]. These limitations restrict the clinical translation of AI-based predictive tools and hinder their adoption across diverse medical settings [19].

Therefore, this systematic review and meta-analysis aims to provide a comprehensive, critical evaluation of the diagnostic performance of CT-based AI technologies in predicting early recurrence of cholangiocarcinoma.

## Methods

We rigorously implemented the Preferred Reporting Items for Systematic Reviews and Meta-Analyses of Diagnostic Test Accuracy Studies (PRISMA-DTA) protocol to ensure comprehensive methodology in our systematic review and meta-analysis [20]. The Preferred Reporting Items for Systematic Reviews and Meta-Analyses (PRISMA) checklist is provided in Multimedia Appendix 1.

### Search Strategy

We comprehensively searched multiple databases (PubMed, Embase, and Web of Science) with an initial cutoff on March 25, 2025, and a supplementary search in May 2025 to ensure inclusion of the latest research. The search strategy incorporated 3 keyword groups: the first group comprised AI-related terms (such as “artificial intelligence,” “radiomic,” and “deep learning”), the second group focused on target-related terms (including “early recurrence” and “tumor recurrence”), and the third group included disease-related terms (such as “cholangiocarcinoma”). We used a combined approach of free-text terms and Medical Subject Headings, with no initial restrictions on language or publication year. Detailed search strategy information is provided in Multimedia Appendix 2. Additionally, we manually reviewed the reference lists of included studies to identify further relevant literature.

### Inclusion and Exclusion Criteria

Studies were included based on the Participants, Index test, Target condition, Reference standard, Outcomes, and Setting (PITROS) framework. For comprehensive details, please consult Textbox 1.

**Summary of inclusion criteria using the Participants, Index test, Target condition, Reference standard, Outcomes, and Setting framework.**

**Participants**

- Patients diagnosed with cholangiocarcinoma, covering both intrahepatic and extrahepatic anatomical locations, were included in this study.

**Index test**

- Artificial intelligence techniques were applied to analyze computed tomography imaging to predict early recurrence of cholangiocarcinoma (defined as within 1 year).

**Target condition**

- The target condition was early recurrence of cholangiocarcinoma, with the positive group defined as patients who developed early recurrence and the negative group as those without early recurrence.

**Reference standard**

- Pathological biopsy or clinical imaging follow-up was used as the reference standard to confirm recurrence status.

**Outcomes**

- The primary outcomes were sensitivity, specificity, diagnostic odds ratio, and area under the receiver operating characteristic curve, evaluated in both internal and external validation cohorts.

**Setting**

- Studies with retrospective or prospective designs were considered, utilizing data from local hospitals.

Additionally, we systematically excluded studies with obviously irrelevant titles and abstracts, as well as inappropriate literature types, including reviews, conference abstracts, case reports, meta-analyses, and letters to editors. Furthermore, non-English studies were excluded due to inaccessibility. Moreover, articles that did not explicitly specify CT-based methodologies and those from which true positive (TP), false positive (FP), true negative (TN), and false negative (FN) values could not be obtained were also excluded.

### Quality Assessment

We used an improved version of the revised Quality Assessment of Diagnostic Accuracy Studies-2 (QUADAS-2) assessment protocol to systematically evaluate the methodological characteristics of the studies [21]. QUADAS-2 is specifically designed to assess bias risk and applicability in diagnostic studies, with its earlier versions having been widely applied across various research contexts. However, we identified certain irrelevant criteria that were not applicable in our context and incorporated the Prediction Model Risk of Bias Assessment Tool (PROBAST) to improve and adapt the risk of bias assessment standards for predictive models [22]. The modified QUADAS-2 tool encompassed 4 primary domains: participants, index test (ie, the applied AI algorithm), reference standard, and analysis. Detailed scoring criteria are presented in Multimedia Appendix 3. Moreover, for certainty rating, the Grading of Recommendations, Assessment, Development, and Evaluations (GRADE) tool was used to assess the evidence level for each standard [23], with scoring details provided in Multimedia Appendix 4. To ensure the objectivity and accuracy of the assessment process, 2 independent reviewers (JC and JX) comprehensively evaluated the bias risk of included studies. During the review process, any disagreements between reviewers were resolved through in-depth discussion and analysis.

### Data Extraction

The 2 reviewers (JC and JX) independently conducted preliminary screenings of the remaining articles’ titles and abstracts to determine their potential eligibility. In cases of disagreement, a third reviewer (KL) would serve as a supervisor to assist in reaching a consensus. The extracted data included patient and study-level information (first author’s name, publication year, patient origin country, type of cholangiocarcinoma, reference standard, lesion-based or patient-based analysis, training set, and total number of patients in internal and external validation sets). Imaging technology–level information included CT type, AI method, AI algorithm, data segmentation method, and TP, TN, FP, and FN values in training, internal, and external validation sets. TP referred to the outcome where the AI model judged as positive based on CT and confirmed as early recurrence of cholangiocarcinoma through pathology or clinical imaging follow-up. TN referred to the AI model judging as negative based on CT and the reference standard confirming the absence of cholangiocarcinoma (ie, no disease present). FP indicated the AI model judged as positive based on CT but confirmed as negative by reference standard (eg, misdiagnosed as a disease but actually a benign lesion or normal variation). FN represented cases where the AI model judged as negative based on CT but confirmed as positive by reference standard (ie, missed disease cases). For studies included in the systematic review but lacking data available for meta-analysis, we sent emails to corresponding authors to obtain the necessary information.

Given the limited availability of diagnostic contingency tables in the majority of studies, we implemented 2 primary methodological approaches to construct 4-cell diagnostic matrices. First, we retroactively calculated TP, FP, TN, and FN using sensitivity, specificity, and the number of positive cases under the reference standard and total patient numbers. Second, through receiver operating characteristic curve analysis, we used GetData Graph Digitizer software (S Fedorov) to replot points, extracting the optimal sensitivity and specificity based on the best Youden index, and then retroactively calculated TP, FP, TN, and FN in combination with the number of positive cases under the reference standard and total patient numbers. In studies providing multiple nonoverlapping patient datasets, we assumed these contingency tables to be independent and therefore extracted them in full. For studies presenting AI models with multiple algorithms, we also performed a comprehensive extraction to enhance the feasibility of comparing algorithms between different approaches.

### Outcome Measures

The primary outcome measures included sensitivity, specificity, diagnostic odds ratio (DOR), and AUC from both internal and external validation sets. Sensitivity (also known as recall or TP rate) measures the ability of the AI model to accurately identify true cases, calculated by the formula TP/(TP+FN). Specificity (also known as TN rate) reflects the model’s capability to correctly identify healthy cases, calculated by the formula TN/(TN+FP). AUC, a comprehensive indicator for evaluating the model’s ability to distinguish between positive and negative cases. DOR is a comprehensive diagnostic performance metric that combines sensitivity and specificity. This metric quantifies the diagnostic test’s discriminative capability by comparing the probability of a positive result in populations with disease versus without disease.

### Statistical Analysis

Using a bivariate random-effects statistical framework, we comprehensively analyzed the diagnostic capabilities of AI applied to CT imaging for early cholangiocarcinoma recurrence detection. We separately summarized diagnostic results for internal and external validation sets, calculating pooled sensitivity, specificity, and DOR values. Forest plots showed combined sensitivity and specificity, and a summary receiver operating characteristic curve illustrated the 95% confidence and prediction ranges, with pooled AUC values calculated.

To assess interstudy heterogeneity, we used the Higgins *I*² statistic, where *I*² values of 25%, 50%, and 75% represent low, moderate, and high heterogeneity, respectively. For substantial heterogeneity (*I*²>50%), we conducted subgroup analysis and meta-regression to explore potential sources of heterogeneity. Included subgroup analysis and meta-regression variables comprised type of cholangiocarcinoma, analysis type, reference standard, type of CT, AI method, AI model, and data splitting method. Additionally, we used bubble plots to assess the variation of AI model DOR values over time. We also used Fagan plots to evaluate the clinical application impact of AI models. Furthermore, we used Deeks’ funnel plot asymmetry test, assessing publication bias through effective sample size and DOR logarithmic regression. Statistical analyses were performed with Midas in Stata (version 15.1; StataCorp LLC), and risk of bias was assessed using RevMan (version 5.4; Cochrane). All tests were 2-sided, with *P*<.05 considered significant.

## Results

### Study Selection

**Figure 1:**
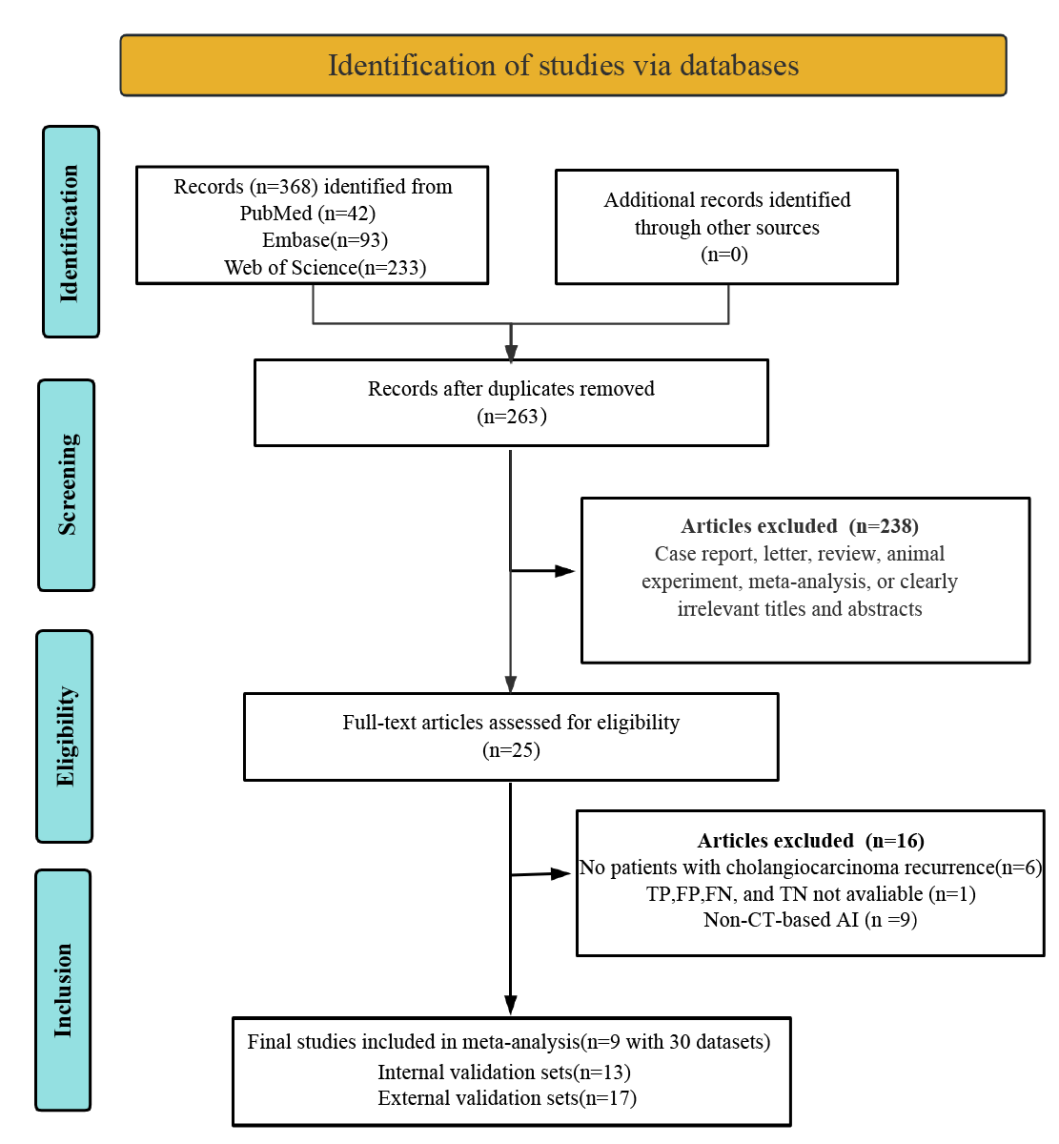
PRISMA (Preferred Reporting Items for Systematic Reviews and Meta-Analyses) flow diagram of study selection for the diagnostic performance of computed tomography (CT)–based artificial intelligence (AI) in early cholangiocarcinoma recurrence. FN: false negative; FP: false positive; TN: true negative; TP: true positive.

A total of 3 initial database searches discovered 368 potentially relevant literatures. After removing 105 duplicate articles, 263 articles entered the preliminary screening phase. During the initial screening, 238 articles were excluded due to irrelevant titles and abstracts and inappropriate literature types, leaving 25 articles for full-text review. After detailed examination, we excluded 1 study with insufficient or incomplete diagnostic data (including TPs, FPs, FNs, and TNs), 9 studies not based on CT-based AI models, and 6 studies that failed to evaluate cholangiocarcinoma recurrence in patients. Ultimately, 9 studies met the inclusion criteria and were incorporated into the meta-analysis [17,24-31]. The literature screening process followed the standardized PRISMA protocol, which is detailed in Figure 1.

### Study Description and Quality Assessment

A total of 9 eligible studies were identified, with internal validation sets comprising 8 studies and 13 datasets [17,25-31], encompassing 1234 patients. External validation involved 5 studies with 17 datasets [24,26,27,29,30], totaling 303 patients. These included studies were published between 2021 and 2023. A total of 78% (7/9) of the studies were conducted in Asia (Japan=1 and China=6), with the remaining 22% (2/9) in North America (United States=2). All 9 studies were retrospective. Overall, 5 studies used pathology and clinical imaging follow-up as the reference standard [24,26,27,29,31], while 4 studies used clinical imaging follow-up alone as the reference standard [17,25,28,30]. Additionally, 8 studies focused on IHC [17,24-28,30,31], while 1 study addressed EHC [29]. Furthermore, 8 studies conducted patient-based analysis [24-31], and 1 study performed image-based analysis [17]. Regarding data splitting methods, 4 studies used random splitting [28-31], 4 studies used cross-validation [17,25-27], and 1 study divided data based on 2 independent hospitals [24]. In terms of AI approaches, 8 studies used machine learning [24-31] and 1 study used deep learning [17]. For internal validation sets, the most frequently used modeling methods were random forest (2/13, 15%) and logistic regression (2/13, 15%). For external validation sets, the most common modeling methods included light gradient boosting machine (LightGBM; 3/17, 18%), logistic regression (2/17, 12%), random forest (2/17, 12%), neural network (2/17, 12%), Bayesian classifier (2/17, 12%), support vector machine (2/17, 12%), and extreme gradient boosting (XGBoost; 2/17, 12%). Study, patient, and technical characteristics are summarized in Table 1 and Multimedia Appendix 5.

**Table 1:** Study and patient characteristics of the included studies.

Reference	Country	Study design	Type of CCA^a^	Reference standard	Analysis	Patients, lesions, or images per set, n				Early recurrence (patients, lesions, or images), n		
						Training	Internal validation	External validation	Training		Internal validation	External validation
Hao et al (2021) [27]	China	Retro^b^	IHC^c^	Pathology and clinical imaging follow-up	PB^d^	124	124	53	N/A^e^		66	35
Song et al (2023) [30]	China	Retro	IHC	Clinical imaging follow-up	PB	140	36	135	75		19	71
Wakiya et al (2022) [17]	Japan	Retro	IHC	Clinical imaging follow-up	IB^f^	71,081	71,081	N/A	N/A		45,316	N/A
Jolissaint et al (2022) [28]	America	Retro	IHC	Clinical imaging follow-up	PB	97	41	N/A	28		11	N/A
Bo et al (2023) [24]	China	Retro	IHC	Pathology and clinical imaging follow-up	PB	90	N/A	37	49		22	N/A
Qin et al (2021) [29]	China	Retro	EHC^g^	Pathology and clinical imaging follow-up	PB	167	70	37	84		46	30
Chen et al (2023) [26]	China	Retro	IHC	Pathology and clinical imaging	PB	95	95	41	N/A		31	13
Zhu et al (2021) [31]	China	Retro	IHC	Pathology and clinical imaging	PB	92	33	N/A	24		11	N/A
Chakraborty et al (2022) [25]	America	Retro	IHC	Clinical imaging follow-up	PB	139	139	N/A	N/A		39	N/A

^a^CCA: cholangiocarcinoma.

^b^Retro: retrospective.

^c^IHC: intrahepatic cholangiocarcinoma.

^d^PB: patient-based.

^e^N/A: not available.

^f^IB: image-based.

^g^EHC: extrahepatic cholangiocarcinoma.

**Figure 2:**
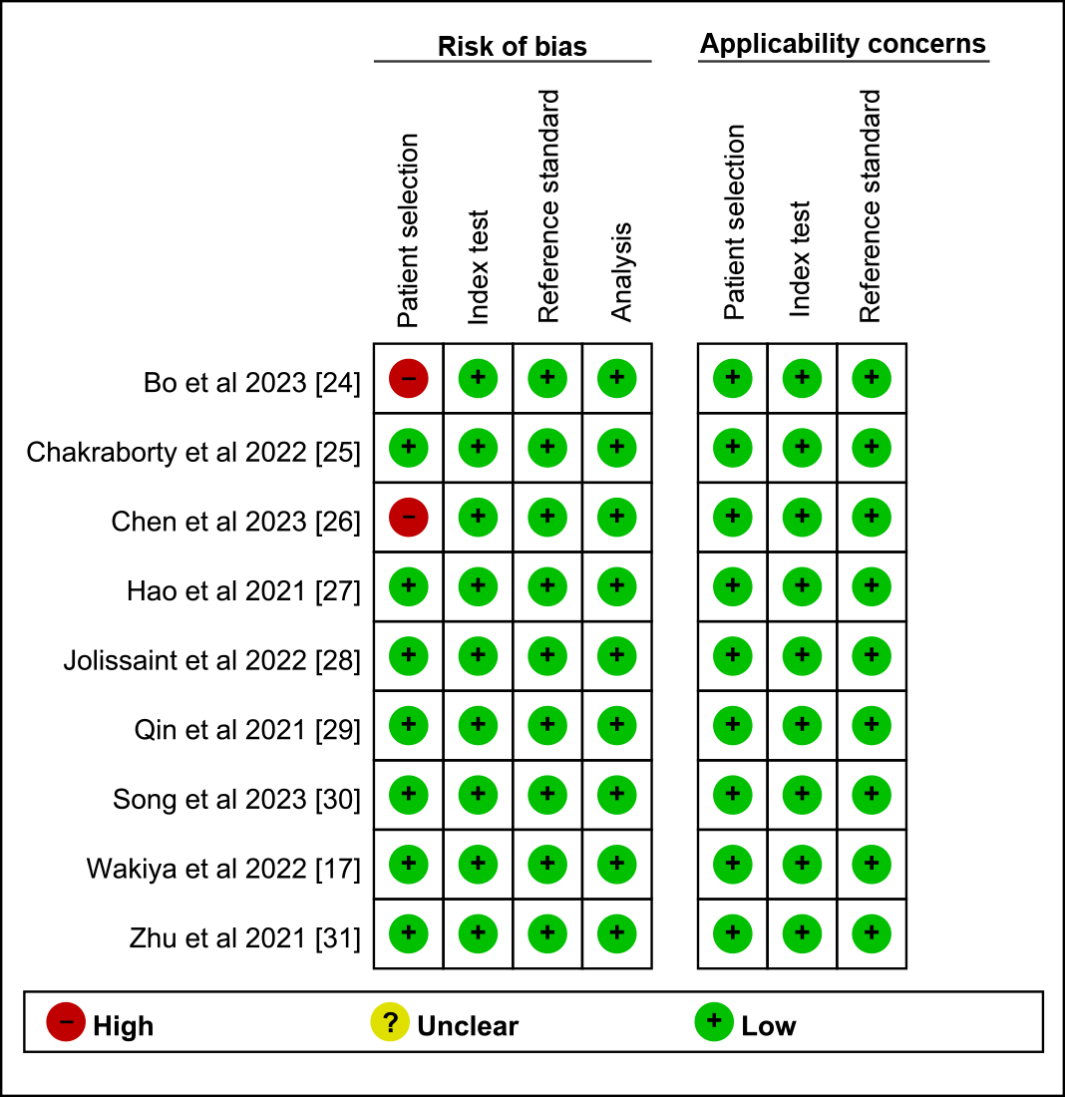
Risk of bias and applicability concerns of the included studies using the revised Quality Assessment of Diagnostic Performance Studies-2 [17,24-31].

Bias risk was assessed using the revised QUADAS-2 tool, as shown in Figure 2. In patient selection, 2 studies were rated as “high risk.” Bo et al [24] inappropriately excluded patients with Child-Pugh scores >7, and Chen et al [26] excluded patients with performance status scores >2 or Child-Pugh scores >7. Overall, high-risk items were few, with low-risk items predominantly represented, indicating that the included studies were acceptable in overall quality (Multimedia Appendix 3). According to GRADE standards, the evidence quality assessment for outcome indicators ranged from very low to low, suggesting weak certainty of evidence (Multimedia Appendix 4).

### Diagnostic Performance of Different AI Algorithms in Internal and External Validation Sets

**Figure 3:**
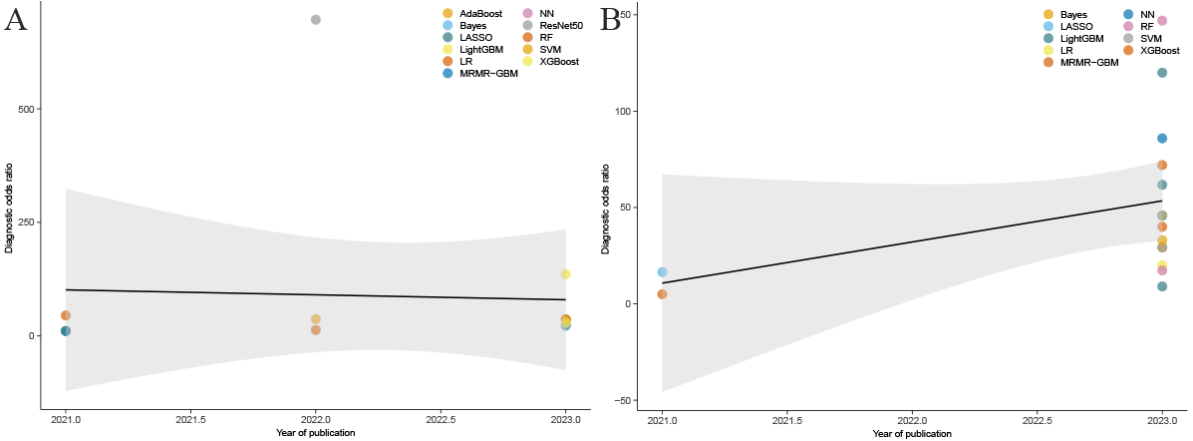
Bubble plot showing diagnostic odds ratios of various artificial intelligence algorithms for cholangiocarcinoma recurrence prediction across publication years. (A) Bubble plot of diagnostic odds ratios for the internal validation cohort. (B) Bubble plot of diagnostic odds ratios for the external validation cohort. AdaBoost: adaptive boosting; Bayes: Bayesian classifier; LASSO: least absolute shrinkage and selection operator; LightGBM: light gradient boosting machine; LR: logistic regression; MRMR-GBM: minimum redundancy maximum relevance-gradient boosting machine; NN: neural network; ResNet50: residual network 50; RF: random forest; SVM: support vector machine; XGBoost: extreme gradient boosting.

Emerging computational intelligence technologies, particularly machine learning and deep learning, have demonstrated remarkable progress through enhanced algorithmic designs and increased data accessibility. In internal validation sets, the DOR remained relatively stable from 2021 to 2023, with some algorithms (residual network 50 [ResNet50] and XGBoost) achieving higher DOR values, while others maintained lower levels (Figure 3A). In external validation sets, DOR values showed a slow increase during the same period, with some algorithms (random forest and LightGBM) achieving higher DOR values, suggesting potential improvements or refinements in AI algorithms (Figure 3B).

In internal validation sets, ResNet50 simultaneously achieved the highest sensitivity (0.98) and specificity (0.94). In external validation sets, the neural network demonstrated the highest sensitivity (0.94), while the support vector machine showed the highest specificity (0.88), as detailed in Table 2.

**Table 2:** Subgroup analysis based on different artificial intelligence (AI) algorithms.

	Interval validation				External validation			
AI algorithms	Studies, n (%)	Sensitivity (95% CI)	Specificity (95% CI)	Studies, n (%)		Sensitivity (95% CI)	Specificity (95% CI)	
LR^a^	2 (15.4)	0.79 (0.64-0.88)	0.88 (0.80-0.94)	2 (11.8)		0.89 (0.73-0.96)	0.81 (0.67-0.90)	
RF^b^	2 (15.4)	0.88 (0.72-0.93)	0.78 (0.68-0.55)	2 (11.8)		0.83 (0.67-0.92)	0.86 (0.72-0.94)	
MRMR-GBM^c^	1 (7.7)	0.88 (0.78-0.95)	0.60 (0.47-0.73)	1 (5.9)		0.71 (0.54- 0.85)	0.67 (0.41- 0.87)	
LightGBM^d^	1 (7.7)	0.89 (0.67-0.99)	0.94 (0.71-1.00)	3 (17.6)		0.92 (0.85-0.96)	0.76 (0.65-0.84)	
ResNet50^e^	1 (7.7)	0.98 (0.98-0.98)	0.94 (0.94-0.94)	N/A^f^		N/A	N/A	
LASSO^g^	1 (7.7)	0.74 (0.59-0.86)	0.79 (0.58-0.93)	1 (5.9)		0.73 (0.54-0.88)	0.86 (0.42-1.00)	
NN^h^	1 (7.7)	0.84 (0.66-0.95)	0.88 (0.77-0.94)	2 (11.8)		0.94 (0.80-0.99)	0.81 (0.67-0.90)	
Bayes^i^	1 (7.7)	0.87 (0.70-0.96)	0.77 (0.64-0.86)	2 (11.8)		0.83 (0.67-0.92)	0.86 (0.72-0.94)	
SVM^j^	1 (7.7)	0.84 (0.66-0.95)	0.88 (0.77-0.94)	2 (11.8)		0.83 (0.67-0.92)	0.88 (0.75-0.95)	
XGBoost^k^	1 (7.7)	0.81 (0.63-0.93)	0.88 (0.77-0.94)	2 (11.8)		0.91 (0.77-0.97)	0.84 (0.70-0.92)	
AdaBoost^l^	1 (7.7)	0.82 (0.66-0.92)	0.89 (0.81-0.94)	N/A		N/A	N/A	

^a^LR: logistic regression.

^b^RF: random forest.

^c^MRMR-GBM: minimum redundancy maximum relevance-gradient boosting machine.

^d^LightGBM: light gradient boosting machine.

^e^ResNet50: residual network 50.

^f^N/A: not available.

^g^LASSO: least absolute shrinkage and selection operator.

^h^NN: neural network.

^i^Bayes: Bayesian classifier.

^j^SVM: support vector machine.

^k^XGBoost: extreme gradient boosting.

^l^AdaBoost: adaptive boosting.

### Diagnostic Performance of CT-Based AI Models for Early Recurrence of Cholangiocarcinoma in Internal Validation Sets

**Figure 4:**
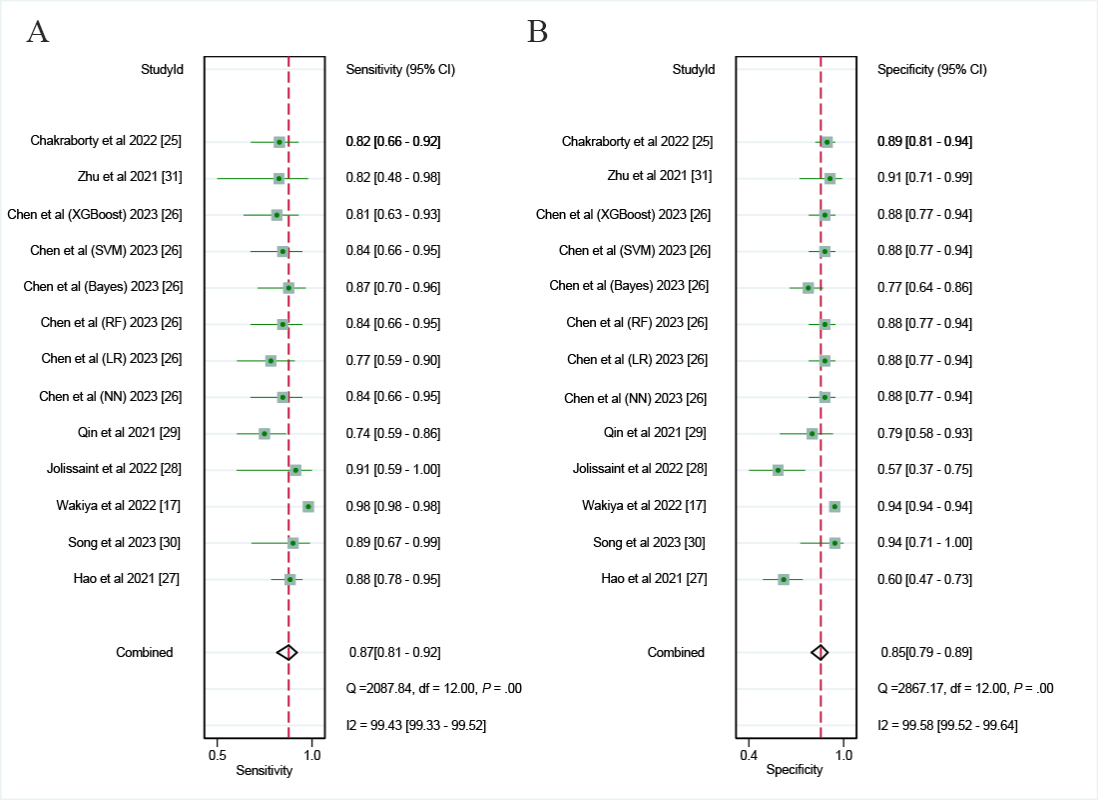
Forest plots of the diagnostic odds ratio of computed tomography–based artificial intelligence on the (A) internal validation set and (B) external validation set for diagnosing early recurrence of cholangiocarcinoma [17,24-31]. Bayes: Bayesian classifier; LR: logistic regression; NN: neural network; RF: random forest; SVM: support vector machine; XGBoost: extreme gradient boosting.

**Figure 5:**
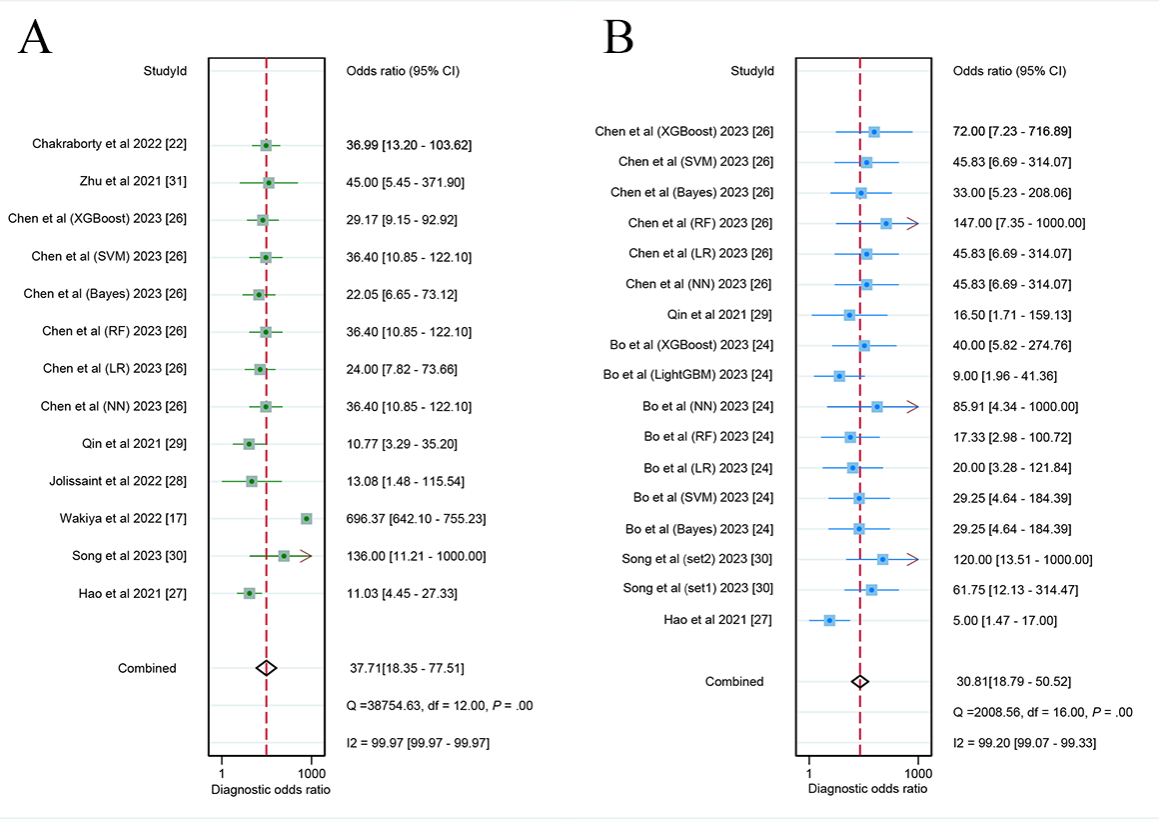
Forest plots of the diagnostic odds ratio of computed tomography–based artificial intelligence on the (A) internal validation set and (B) external validation set for diagnosing early recurrence of cholangiocarcinoma. CI [17,24-31]. Bayes: Bayesian classifier; LR: logistic regression; NN: neural network; RF: random forest; SVM: support vector machine; XGBoost: extreme gradient boosting.

**Figure 6:**
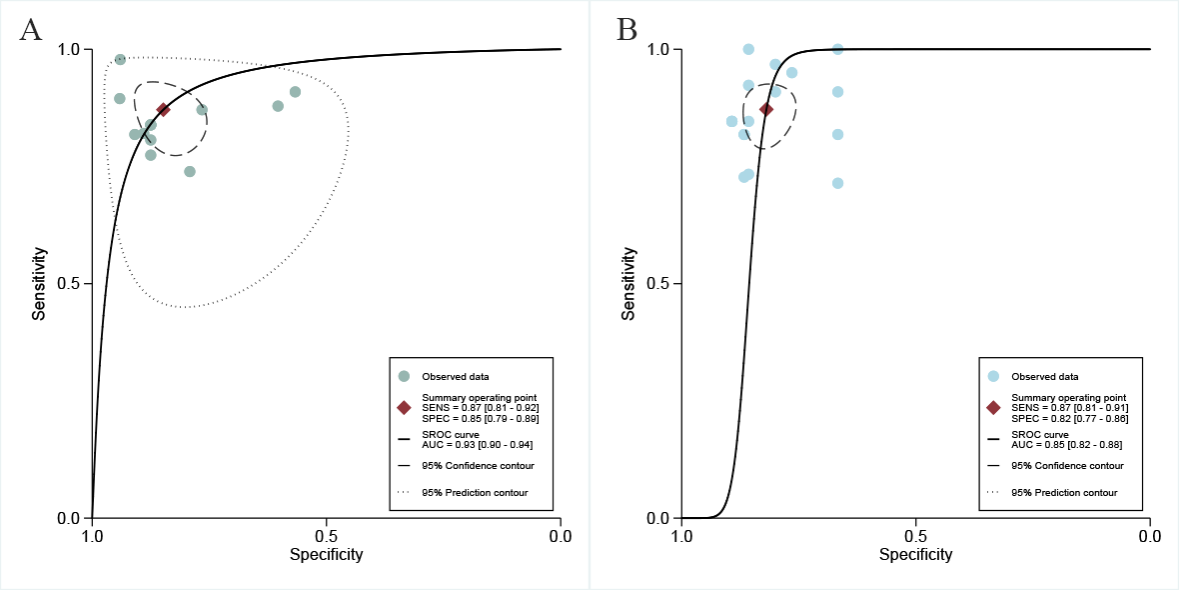
Summary receiver operating characteristic (SROC) curves of computed tomography–based artificial intelligence on the (A) internal validation set and (B) external validation set for predicting early recurrence of cholangiocarcinoma. AUC: area under the receiver operating characteristic curve; SENS: sensitivity; SPEC: specificity.

In the internal validation sets, the CT-based AI model demonstrated a sensitivity of 0.87 (95% CI 0.81-0.92; very low certainty), specificity of 0.85 (95% CI 0.79-0.89; very low certainty), and a DOR of 37.71 (95% CI 18.35-77.51; very low certainty), as shown in Figures 4 and 5A. Additionally, the AUC of the model was 0.93 (95% CI 0.90-0.94; Figure 6A). Based on the predefined 20% test probability, the Fagan nomogram revealed a positive likelihood ratio of 59% and a negative likelihood ratio of 4% (Multimedia Appendix 6).

### Meta-Regression and Subgroup Analysis in Internal Validation Sets

Meta-regression revealed high heterogeneity in sensitivity (*I*²=99.43%) and specificity (*I*²=99.58%) for internal validation sets. The meta-regression analysis results showed no identifiable sources of potential heterogeneity (Table 2). Subgroup analysis demonstrated no statistically significant differences in sensitivity and specificity of CT-based AI models across various categories, including type of cholangiocarcinoma, analysis, reference standard, AI model, AI method, and data splitting method (all *P*>.05; Table 3).

**Table 3:** Subgroup analysis and meta-regression analysis of the diagnostic performance of computed tomography (CT)–based artificial intelligence (AI) for early recurrence of cholangiocarcinoma within internal validation cohorts.

Subgroup		Studies, n (%)	Sensitivity (95% CI)	Meta-regression, *P* value		Specificity (95% CI)		Meta-regression, *P* value	
**Type of cholangiocarcinoma**					.13				.49
	IHC^a^	12 (92.3)	0.88 (0.83-0.93)			0.85 (0.80-0.90)			
	PHC^b^	1 (7.7)	0.74 (0.46-1.00)			0.80 (0.54-1.00)			
**Analysis**					.91				.22
	Lesion-based	1 (7.7)	0.83 (0.60-1.00)			0.89 (0.76-1.00)			
	Patient-based	12 (92.3)	0.87 (0.82-0.93)			0.84 (0.79-0.90)			
**Reference standard**					.57				.57
	Pathology and clinical imaging follow-up	9 (69.2)	0.83 (0.77-0.90)			0.84 (0.77-0.91)			
	Clinical imaging follow-up	4 (30.8)	0.94 (0.90-0.99)			0.87 (0.79-0.95)			
**Type of CT**					.47				.87
	CECT^c^	12 (92.3)	0.87 (0.82-0.93)			0.84 (0.79-0.90)			
	Plain CT	1 (7.7)	0.83 (0.60-1.00)			0.89 (0.76-1.00)			
**AI model**					.35				.56
	Radiomic model	2 (15.4)	0.86 (0.72-1.00)			0.78 (0.62-0.94)			
	Radiomic and clinical model	11 (84.6)	0.87 (0.81-0.93)			0.86 (0.81-0.91)			
**AI method**					.95				.16
	Deep learning	1 (7.7)	0.92 (0.73-1.00)			0.57 (0.26-0.87)			
	Machine learning	12 (92.3)	0.87 (0.82-0.93)			0.86 (0.82-0.90)			
**Data splitting method**					.32				.22
	Random split	4 (30.8)	0.84 (0.71-0.96)			0.81 (0.68-0.94)			
	K-fold cross-validation	9 (69.2)	0.88 (0.83-0.94)			0.86 (0.80-0.91)			

^a^IHC: intrahepatic cholangiocarcinoma.

^b^PHC: perihilar cholangiocarcinoma.

^c^CECT: contrast-enhanced computed tomography.

### Diagnostic Performance of CT-Based AI Models for Early Cholangiocarcinoma Recurrence in External Validation Sets

In the validation sets conducted externally, the AI model based on CT exhibited a sensitivity of 0.87 (95% CI 0.81-0.91; low certainty) and a specificity of 0.82 (95% CI 0.77-0.86; very low certainty; Multimedia Appendix 7). Additionally, it showed a DOR of 30.81 (95% CI 18.79-50.52; very low certainty; Figure 5B) and an AUC measuring 0.85 (95% CI 0.82-0.88; Figure 6B). When applying a 20% pretest probability, the Fagan nomogram indicated a positive likelihood ratio of 55% along with a negative likelihood ratio of 4% (Multimedia Appendix 6).

No statistically significant variances were found between the internal and external validation sets regarding sensitivity (*z* score=0.00; *P*>.99), specificity (*z* score=0.87; *P*=.38), and DOR (*z* score=0.00; *P*=.08). However, the AUC for the internal validation set was significantly greater than that of the external validation set (*z* score=4.35; *P*<.001).

### Meta-Regression and Subgroup Analysis in External Validation Sets

Meta-regression revealed high heterogeneity in sensitivity (*I*²=42.07%), and although specificity (*I*²=0%) did not show high heterogeneity, we attempted to identify potential sources of heterogeneity. Meta-regression analysis results indicated that different reference standards (specificity, *P*<.001) and type of cholangiocarcinoma (sensitivity, *P*=.05) might be potential sources of heterogeneity. In the cholangiocarcinoma-type subgroups, the sensitivity was 0.88 (95% CI 0.83-0.92) for IHC and 0.74 (95% CI 0.50-0.98) for EHC, with IHC showing significantly higher sensitivity compared to EHC (*P*=.05). In the recurrence reference standard subgroups, the specificity of pathology combined with clinical imaging follow-up was 0.83 (95% CI 0.78-0.87), which was significantly higher than that of clinical imaging follow-up alone (0.78, 95% CI 0.68-0.88; *P*<.001; Multimedia Appendix 8).

### Sensitivity Analysis and Bivariate Box Plot

For the internal validation sets, after excluding low-quality studies, the AI model’s sensitivity was 0.90 (95% CI 0.80-0.95), specificity was 0.84 (95% CI 0.71-0.91), DOR was 43.43 (95% CI 12.31-153.31), and AUC was 0.93 (95% CI 0.91-0.95). For the external validation sets, after excluding low-quality studies, the AI model’s sensitivity was 0.88 (95% CI 0.70-0.96), specificity was 0.76 (95% CI 0.44-0.84), DOR was 21.79 (95% CI 5.74-82.68), and AUC was 0.80 (95% CI 0.77-0.84; Multimedia Appendix 9).

For the internal validation sets, the bivariate box plot suggested that Wakiya et al [17], Song et al [30], and Zhu et al [31] may represent potential contributors to statistical heterogeneity. After excluding the studies identified in the bivariate box plot, the AI model’s sensitivity was 0.83 (95% CI 0.78-0.87), specificity was 0.82 (95% CI 0.75-0.88), DOR was 22.28 (95% CI 14.43-34.40), and AUC was 0.87 (95% CI 0.84-0.90). For the external validation sets, the bivariate box plot suggested that Chen et al [26] and Hao et al [27] might be potential sources of heterogeneity (Multimedia Appendix 10). After excluding the studies identified in the bivariate box plot, the AI model’s sensitivity was 0.88 (95% CI 0.82-0.93), specificity was 0.80 (95% CI 0.74-0.85), DOR was 30.48 (95% CI 17.27-53.79), and AUC was 0.87 (95% CI 0.84-0.90).

### Publication Bias

**Figure 7:**
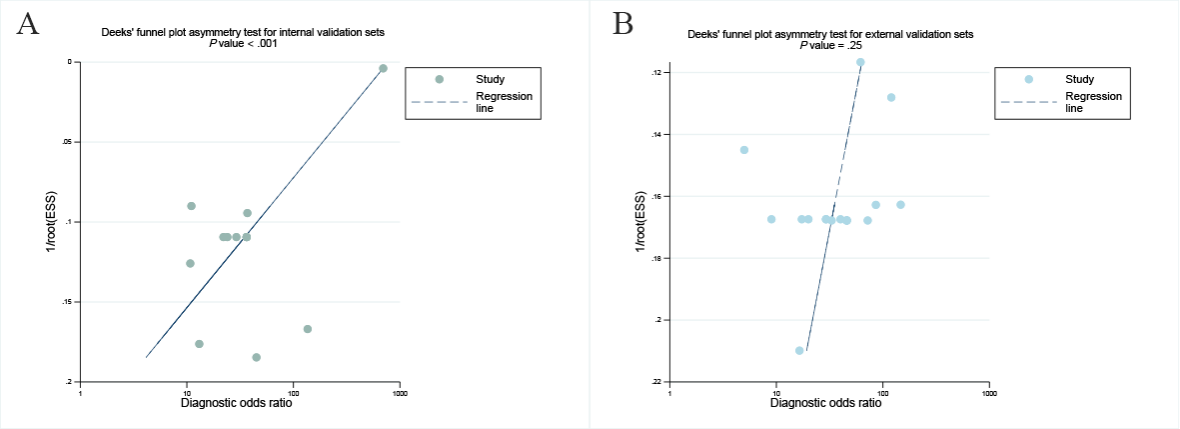
Deek’s funnel plot was used to evaluate the publication bias of computed tomography–based artificial intelligence. *P*<.05 was considered significant.

Using Deeks’ funnel plot methodology, we identified substantial publication bias in the internal validation cohorts (*P*<.001), whereas for the external validation sets, there was no evidence of small-study effects (*P*=.25; Figures 7A-7B).

## Discussion

### Principal Findings

Our study demonstrated the exceptional performance of CT-based AI models in diagnosing early recurrence of cholangiocarcinoma. In the internal validation sets, the model exhibited high diagnostic performance (sensitivity=0.87, specificity=0.85, DOR=37.71, and AUC=0.93). However, a moderate decline in performance was observed during external validation (sensitivity=0.87, specificity=0.82, DOR=30.81, and AUC=0.85), with a statistically significant reduction in AUC (*P*<.001). This finding suggests that despite the model’s excellent performance in internal datasets, its generalizability across different patient populations and imaging conditions may be limited. The outstanding performance of the CT-based AI model primarily stems from its powerful feature extraction capabilities, enabling it to capture high-dimensional radiomics features that reflect tumor heterogeneity and microenvironmental characteristics [32]. These features are particularly crucial for highly invasive and heterogeneous cholangiocarcinoma, which are often challenging to detect through conventional imaging techniques. By using advanced algorithms, such as machine learning and radiomics, the model can effectively identify complex patterns within imaging data, thereby enabling precise prediction of early recurrence risk in cholangiocarcinoma [29,33,34]. However, the performance decline observed in external validation highlighted several potential limitations. First, heterogeneity in CT acquisition techniques across different institutions (such as scanner models, contrast agent protocols, and reconstruction parameters) may compromise the model’s stability [35]. Second, variability in patient demographics and pathological characteristics may further contribute to decreased performance in external cohorts. These findings underscore the importance of conducting large-scale, multicenter external validation studies to ensure the robustness and generalizability of AI models across different clinical environments [36].

Our subgroup analysis results based on different cholangiocarcinoma subtypes revealed that in the external validation sets, the sensitivity of IHC was significantly higher than that of EHC (*P*=.05). A similar trend was observed in the internal validation sets, although the difference was not statistically significant. This difference may be attributed to the distinct biological characteristics and imaging features of IHC and EHC. IHC typically demonstrates pronounced tumor heterogeneity and microenvironmental characteristics, which are more readily captured by AI algorithms on CT imaging, thereby enhancing diagnostic sensitivity. In contrast, the anatomical complexity of EHC and its less distinctive imaging characteristics may limit the diagnostic performance of AI models [37,38]. It is important to note that the EHC dataset was limited (only 1 group of data), which may constrain the model’s learning capacity for EHC. Future multicenter studies are needed to validate these findings.

### Comparison to Previous Work

In 2023, Yang et al [39] conducted a meta-analysis evaluating the diagnostic performance of machine learning models for early recurrence of IHC. The analysis included 5 studies comprising a total of 1247 patients, using paired and network meta-analysis to compare the diagnostic accuracy of machine learning models with traditional clinical models. The results demonstrated that machine learning models exhibited a pooled diagnostic sensitivity of 0.92, a specificity of 0.79, and an AUC of 0.81, revealing superior diagnostic value compared to traditional clinical models. In contrast, our AI model demonstrated higher diagnostic performance for predicting early recurrence of cholangiocarcinoma in the internal validation set, with an AUC value of 0.93. This may be attributed to the larger sample size included in our study, as well as the use of more advanced deep learning algorithms. However, it is important to note that, in contrast to the previous meta-analysis, our study focused solely on CT-based AI models and used a more specific data source for model training.

In 2025, Xu et al [40] conducted a meta-analysis evaluating the application of machine learning based on radiomics in IHC. Their results showed that AI diagnostic models integrating radiomics and clinical features achieved a sensitivity of 0.85 and specificity of 0.77. Our study showed that the CT-based AI model achieved a sensitivity of 0.87 and a specificity of 0.85. Compared to previous research, our diagnostic performance was superior, which may be related to differences in patient populations and AI models included. Xu et al [40] focused primarily on IHC cases, whereas our study included both IHC and EHC. Additionally, our analysis exclusively evaluated CT-based AI models. Compared with previous meta-analyses, our unique strength lies in merging different specific algorithms separately and stratifying the study population into internal and external validation sets to assess the generalizability of the AI models, thus providing more rigorous and comprehensive diagnostic evidence.

### Heterogeneity

The high heterogeneity of included studies might affect the overall sensitivity and specificity of AI models in internal and external validation sets. For internal validation sets, we sought to identify sources of heterogeneity through meta-regression and box plot analysis. While meta-regression did not identify any significant factors, box plots indicated that studies by Wakiya et al [17], Zhu et al [31], and Song et al [30] might be primary sources of heterogeneity. In the external validation sets, meta-regression indicated that cholangiocarcinoma types and reference standards were potential sources of heterogeneity, while box plots also identified studies by Chen et al [26] and Hao et al [27] as significant contributors to heterogeneity. Nevertheless, high heterogeneity may still result from multiple interacting factors, including patient age and status, tumor stage, geographical region, preprocessing methods, classification algorithms and validation approaches, sample size, and AI model characteristics, such as feature selection, hyperparameter optimization, and modeling algorithms [41-44]. The complex interplay of these factors may cause differences between studies, highlighting the need for future research to standardize variables and reduce heterogeneity for more accurate and reliable results. To address the challenges posed by heterogeneity, future studies should consider implementing harmonization techniques, which standardize imaging parameters and processes used across different institutions. Robust data augmentation methods can enhance the diversity of training datasets, enabling models to generalize effectively across diverse populations. Additionally, domain adaptation strategies can facilitate the transfer of learned features from source datasets to new, unseen populations, thereby enhancing model robustness and generalizability [45].

### Future Directions

Our results demonstrate that CT-based AI models achieve high diagnostic performance in internal validation sets and moderate performance in external validation sets. AI can perform preliminary reading, enabling clinicians to process cases more quickly, improve turnaround time, expand the accessibility of specialized reports, and ultimately alleviate pressure on the health care system. Implementing CT-based AI models in primary health care systems, such as general practice, may lead to early disease detection and timely intervention. However, it is worth emphasizing that these models should not be viewed as independent standards or decision-making tools, but rather as useful resources in emergency situations (when expert consultation is unavailable) or for residents and clinicians lacking expertise in detecting this disease. In addition, none of the studies included in our research reported the comparative performance of AI-assisted human interpretation against that of AI alone, making it challenging to assess the specific added value of AI tools for human readers. Additionally, the studies did not detail how AI models perform relative to radiologists, which may limit the practical interpretability of the results. Future research could benefit from exploring comparative analyses that examine the interplay between AI and radiologists. Moreover, while our study primarily focused on AI models based on CT imaging, it is important to acknowledge the inherent limitations of relying solely on this modality. CT imaging does not account for the comprehensive insights that can be gained from MRI and ultrasound imaging techniques. Each of these imaging modalities has unique strengths; for example, MRI excels in soft tissue contrast and provides functional information, while ultrasound offers real-time imaging capabilities and is more accessible in certain clinical settings. Future AI model evaluations should consider integrating cross-modal imaging data, associating observations with patients’ clinical backgrounds, and effectively communicating synthesized insights through reports [46,47].

Additionally, our study primarily focused on traditional machine learning algorithms, with 8 of the 9 included studies using methods like logistic regression, random forests, and support vector machines. Only 1 study used a deep learning approach (ResNet50). Deep learning models typically use image data as their primary input, allowing them to automatically learn hierarchical features from raw data. In contrast, traditional machine learning models rely on parametric values and features that are often manually crafted from the data [48]. Although deep learning excels at handling complex, high-dimensional data, its “black box” nature may result in a lack of interpretability and transparency in clinical settings, which could affect its practical acceptability and the reproducibility of model decisions [49]. Therefore, future research should incorporate a greater number of deep learning models to comprehensively assess their effectiveness and applicability relative to traditional algorithms. At the same time, the importance of explainability in the clinical deployment of AI models cannot be overstated. Future studies should incorporate interpretable frameworks, such as Gradient-Weighted Class Activation Mapping and Shapley Additive Explanations, and attention maps, to ensure that AI-generated predictions can be understood by clinicians [50]. This not only fosters trust in AI systems among health care providers but also facilitates their broader adoption in real-world clinical environments [51].

### Limitations

Our study has several limitations. First, all included studies used retrospective designs, which may introduce potential bias. Well-designed prospective studies are needed to further validate our meta-analysis findings. Second, not all studies defined the gold standard as histopathological examination, which could influence diagnostic performance. However, our subgroup analysis showed no statistically significant differences in sensitivity or specificity among different gold standards, suggesting that the choice of gold standard may have a limited impact. Third, most of the included studies (7/9, 78%) were conducted in Asian regions, which may limit the generalizability of our conclusions.

### Conclusions

Our meta-analysis systematically evaluated the diagnostic performance of CT-based AI models for predicting early recurrence of cholangiocarcinoma. The results indicate that while these models demonstrate high diagnostic accuracy in internal validation cohorts, their performance is only moderate in external validation cohorts. Significant heterogeneity between studies may affect the robustness of these findings. Future research should prioritize prospective study designs and the adoption of standardized reference standards to validate these findings and improve the clinical utility and generalizability of AI models.

## Appendix

Multimedia Appendix 1 PRISMA checklist 2020.

Multimedia Appendix 2 Search strategy in PubMed, Embase, and Web of Science.

Multimedia Appendix 3 Revised Quality Assessment of Diagnostic Accuracy Studies-2 tool for the included studies.

Multimedia Appendix 4 Grading of Recommendations, Assessment, Development, and Evaluations scoring assessments in all of the pooled outcomes.

Multimedia Appendix 5 Technical aspects of included studies.

Multimedia Appendix 6 Fagan’s nomogram for the computed tomography–based artificial intelligence model in predicting early recurrence of cholangiocarcinoma. (A) Internal validation sets. (B) External validation sets.

Multimedia Appendix 7 Forest plots of the combined sensitivity and specificity of computed tomography–based artificial intelligence in patients with early cholangiocarcinoma recurrence. Squares denote the sensitivity and specificity for each study, while horizontal bars indicate the 95% CI.

Multimedia Appendix 8 Subgroup analysis and meta-regression analysis of the diagnostic performance of computed tomography–based artificial intelligence for early recurrence of cholangiocarcinoma within external validation sets.

Multimedia Appendix 9 Sensitivity analysis.

Multimedia Appendix 10 Bivariate boxplot for the computed tomography–based artificial intelligence model in predicting early recurrence of cholangiocarcinoma. (A) Internal validation sets. (B) External validation sets.

## Abbreviations

AI: artificial intelligence
AUC: area under the receiver operating characteristic curve
CT: computed tomography
DOR: diagnostic odds ratio
EHC: extrahepatic cholangiocarcinoma
FN: false negative
FP: false positive
GRADE: Grading of Recommendations, Assessment, Development, and Evaluations
IHC: intrahepatic cholangiocarcinoma
LightGBM: light gradient boosting machine
MRI: magnetic resonance imaging
PITROS: Participants, Index test, Target condition, Reference standard, Outcomes, and Setting
PRISMA: Preferred Reporting Items for Systematic Reviews and Meta-Analyses
PRISMA-DTA: Preferred Reporting Items for Systematic Reviews and Meta-Analyses of Diagnostic Test Accuracy Studies
PROBAST: Prediction Model Risk of Bias Assessment Tool
QUADAS-2: Quality Assessment of Diagnostic Accuracy Studies-2
ResNet50: residual network 50
TN: true negative
TP: true positive
XGBoost: extreme gradient boosting

## Data Availability

The original findings of this study are encompassed within this paper. For additional inquiries, please contact the corresponding authors.
