# Supplementary material for: Diagnostic Performance of Computed Tomography–Based Artificial Intelligence for Early Recurrence of Cholangiocarcinoma: Systematic Review and Meta-Analysis
Source: J Med Internet Res. 2025 Sep 18;27:e78306. doi: 10.2196/78306 (PMC12491900; doi:10.2196/78306)
Supplement: Multimedia Appendix 2 [file jmir_v27i1e78306_app2.docx]

**Table S1.** Search strategy in PubMed, Embase and Web of Science.

| Database | Search strategy |
| --- | --- |
| PubMed | ("Radiomics"[Mesh] OR "Artificial Intelligence"[Mesh] OR "Machine Learning"[Mesh] OR "Deep Learning"[Mesh] OR "Artificial Intelligence"[Title/Abstract] OR "AI"[Title/Abstract] OR "Machine Learning"[Title/Abstract] OR "Deep Learning"[Title/Abstract] OR "Machine Intelligence"[Title/Abstract] OR "Radiomic"[Title/Abstract]) AND ("Neoplasm Recurrence, Local"[Mesh] OR "Recurrence"[Mesh] OR "recurrence*"[Title/Abstract] OR "recurrent"[Title/Abstract] OR "relapse*"[Title/Abstract]) AND ("Cholangiocarcinoma"[Mesh] OR "bile duct cancer"[Title/Abstract] OR "intrahepatic cholangiocarcinoma"[Title/Abstract] OR "extrahepatic cholangiocarcinoma"[Title/Abstract] OR "hilar cholangiocarcinoma"[Title/Abstract] OR "distal cholangiocarcinoma"[Title/Abstract] OR "Klatskin tumor"[Title/Abstract]) |
| Embase | ('radiomics'/exp OR 'artificial intelligence'/exp OR 'machine learning'/exp OR 'deep learning'/exp OR 'artificial intelligence':ab,ti OR 'ai':ab,ti OR 'machine learning':ab,ti OR 'deep learning':ab,ti OR 'machine intelligence':ab,ti OR 'radiomic':ab,ti) AND ('tumor recurrence'/exp OR 'recurrent disease'/exp OR 'recurrence*':ab,ti OR 'recurrent':ab,ti OR 'relapse*':ab,ti ) AND ('bile duct carcinoma'/exp OR 'intrahepatic cholangiocarcinoma'/exp OR 'extrahepatic cholangiocarcinoma'/exp OR 'bile duct cancer':ab,ti OR 'intrahepatic cholangiocarcinoma':ab,ti OR 'extrahepatic cholangiocarcinoma':ab,ti OR 'hilar cholangiocarcinoma':ab,ti OR 'distal cholangiocarcinoma':ab,ti OR 'klatskin tumor':ab,ti ) |
| Web of Science | TS=("Radiomics" OR "Artificial Intelligence" OR "Machine Learning" OR "Deep Learning" OR "Machine Intelligence" OR "Radiomic" OR "Predictive Modeling" OR "AI" OR "Neural Networks" OR "Computer Vision") AND TS=("Neoplasm Recurrence, Local" OR "Recurrence" OR "recurrence*" OR "recurrent" OR "relapse*" OR "Progression" OR "Metastasis" OR "Second Primary" OR "Tumor Recurrence" OR "Disease Recurrence" OR "Cancer Recurrence") AND TS=("Cholangiocarcinoma" OR "bile duct cancer" OR "intrahepatic cholangiocarcinoma" OR "extrahepatic cholangiocarcinoma" OR "hilar cholangiocarcinoma" OR "distal cholangiocarcinoma" OR "Klatskin tumor" OR "Biliary Tract Cancer" OR "Bile Duct Neoplasm" OR "Biliary Carcinoma" OR "Cholangiocellular Carcinoma" OR "Perihilar Cholangiocarcinoma") |
