# Supplementary material for: Diagnostic Performance of Computed Tomography–Based Artificial Intelligence for Early Recurrence of Cholangiocarcinoma: Systematic Review and Meta-Analysis
Source: J Med Internet Res. 2025 Sep 18;27:e78306. doi: 10.2196/78306 (PMC12491900; doi:10.2196/78306)
Supplement: Multimedia Appendix 3 [file jmir_v27i1e78306_app3.docx]

**Table S1.** Revised QUADAS-2 tool for the included studies.

| Author | Risk of bias | | | |  | Applicability concerns | | |
| --- | --- | --- | --- | --- | --- | --- | --- | --- |
|  | Patient selection ^a^ | Index test ^b^ | Reference standard ^c^ | Analysis ^d^ |  | Patient selection ^e^ | Index test ^f^ | Reference standard ^g^ |
| Hao et al.2021 | L | L | L | L |  | L | L | L |
| Song et al.2023 | L | L | L | L |  | L | L | L |
| Wakiya et al.2022 | L | L | L | L |  | L | L | L |
| Jolissaint et al.2022 | L | L | L | L |  | L | L | L |
| Bo et al.2023 | H | L | L | L |  | L | L | L |
| Qin et al.2021 | L | L | L | L |  | L | L | L |
| Chen et al.2023 | H | L | L | L |  | L | L | L |
| Zhu et al.2021 | L | L | L | L |  | L | L | L |
| Chakraborty et al.2022 | L | L | L | L |  | L | L | L |

L low; H high; U unclear.

a. **Patient selection**

- Low risk: No inappropriate exclusions.
- High risk: Inappropriate exclusions (e.g., excluding patients under 18, restricting to specific treatments/subtypes/timeframes).
- Unclear: Insufficient information to assess exclusions.

b. **Index test**

- Low risk: Detailed model training/validation processes provided or cited from a prior publication with full modification details.
- High risk: Only model name reported without key training details (e.g., algorithm unspecified).
- Unclear: Model name given but training process indeterminable.

c. **Reference standard**

- Low risk: Final diagnosis made blinded to AI results.
- High risk: AI results used to inform final diagnosis.
- Unclear: Blinding status unreported.

d. **Analysis**

- Low risk: All enrolled participants included in meta-analysis.
- High risk: Selective exclusion of participants/subgroups.
- Unclear: Inclusion criteria inadequately described.

e. **Patient selection**

- Low risk: Study population aligns with meta-analysis inclusion criteria.
- High risk: Study includes ineligible patients per meta-analysis criteria.
- Unclear: Population eligibility unclear.

f. **Index test**

- Low risk: AI definition matches meta-analysis criteria.
- High risk: AI definition partially deviates from criteria.
- Unclear: AI definition adequacy unverifiable.

g. **Reference standard**

- Low risk: Reference standard aligns with meta-analysis criteria.
- High risk: Reference standard inconsistently applied.
- Unclear: Reference standard details missing.
