# Supplementary material for: Diagnostic Performance of Computed Tomography–Based Artificial Intelligence for Early Recurrence of Cholangiocarcinoma: Systematic Review and Meta-Analysis
Source: J Med Internet Res. 2025 Sep 18;27:e78306. doi: 10.2196/78306 (PMC12491900; doi:10.2196/78306)
Supplement: Multimedia Appendix 4 [file jmir_v27i1e78306_app4.docx]

**Table S1.** GRADE scoring assessments in all of the pooled outcomes.

| Dataset | Outcome | Risk of Bias^a^ | Inconsistency^b^ | Indirectness^c^ | Imprecision^d^ | Publication Bias^e^ | Total Downgrade | Final Rating |
| --- | --- | --- | --- | --- | --- | --- | --- | --- |
| Internal validation dataset | Sensitivity | 1 | 0 | 1 | 0 | 1 | 3 | Very low |
|  | Specificity | 1 | 0 | 1 | 1 | 1 | 4 | Very low |
|  | DOR | 1 | 0 | 1 | 1 | 1 | 4 | Very low |
| External validation dataset | Sensitivity | 1 | 0 | 1 | 0 | 0 | 2 | low |
|  | Specificity | 1 | 0 | 1 | 1 | 0 | 3 | Very low |
|  | DOR | 1 | 0 | 1 | 1 | 0 | 3 | Very low |

a. Risk of Bias

Assessed using tools like QUADAS-2 to evaluate study design and methodology.

Downgrade by 1 level if at least one study has a high risk of bias.

b. Inconsistency

Measured using I² statistics to assess heterogeneity across studies.

Downgrade by 1 level if I² > 50% and the source of heterogeneity cannot be explained.

If the heterogeneity is identified (e.g., through meta-regression), no downgrade is applied.

c. Indirectness

Evaluates whether study populations, interventions, or outcomes differ from the target research question.

Patient Indirectness: Downgrade if the included population significantly deviates from the target population (e.g., specific subgroups).

Outcome Indirectness: Downgrade if inconsistent outcome measures are used, such as estimating sensitivity/specificity indirectly from ROC-based Youden Index.

d. Imprecision

Assessed based on confidence intervals (CIs) and sample size sufficiency.

For sensitivity/specificity: Downgrade if 95% CI width > 0.20 or if CIs cross clinical thresholds (e.g., 0.80).

For DOR: Downgrade if CI range exceeds 1× the lower limit or includes values < 10.

Sample size threshold: Total sample < 500 or positive/negative cases < 100.

e. Publication Bias

Evaluated using Deek’s Funnel Plot or similar tools.

Downgrade by 1 level if the funnel plot shows significant asymmetry or if P-value < 0.05 in Deek’s tests.
