# Supplementary material for: Diagnostic Performance of Computed Tomography–Based Artificial Intelligence for Early Recurrence of Cholangiocarcinoma: Systematic Review and Meta-Analysis
Source: J Med Internet Res. 2025 Sep 18;27:e78306. doi: 10.2196/78306 (PMC12491900; doi:10.2196/78306)
Supplement: Multimedia Appendix 5 [file jmir_v27i1e78306_app5.docx]

**Table S1.** Technical aspects of included studies.

| Author | Year | Imaging | Preprocessing Pipelines | Standardization Methods | AI method | AI model | AI algorithms | Data splitting method | Internal validation sets | | | | External validation sets | | | |
| --- | --- | --- | --- | --- | --- | --- | --- | --- | --- | --- | --- | --- | --- | --- | --- | --- |
|  |  |  |  |  |  |  |  |  | TP | FP | FN | TN | TP | FP | FN | TN |
| Hao et al. | 2021 | CECT | NA | Z-score normalization | Machine learning | Radiomic | MRMR-GBM | 5-fold cross validation | 58 | 23 | 8 | 35 | 25 | 6 | 10 | 12 |
| Song et al.(set1) | 2023 | CECT | Resampling to isotropic 1 × 1 × 1 mm³; CT feature extraction from normalized images; Filtering using Wavelet and Laplacian of Gaussian (LoG) filters | Z-score normalization | Machine learning | Radiomic & clinical model | LightGBM | Random split | 17 | 1 | 2 | 16 | 38 | 8 | 2 | 26 |
| Song et al.(set2) | 2023 | CECT | Resampling to isotropic 1 × 1 × 1 mm³; CT feature extraction from normalized images; Filtering using Wavelet and Laplacian of Gaussian (LoG) filters | Z-score normalization | Machine learning | Radiomic & clinical model | LightGBM | Random split | NA | NA | NA | NA | 30 | 6 | 1 | 24 |
| Wakiya et al. | 2022 | Plain CT | Original image resized from 128 × 128 to 224 × 224 pixels | NA | Deep learning | Radiomic | ResNet50 | 5-fold cross validation | 44,319 | 1,546 | 997 | 24,219 | NA | NA | NA | NA |
| Jolissaint et al. | 2022 | CECT | NA | NA | Machine learning | Radiomic & clinical model | RF | Random split | 10 | 13 | 1 | 17 | NA | NA | NA | NA |
| Bo et al.(set1) | 2023 | CECT | Gray level normalization | Z-score normalization | Machine learning | Radiomic & clinical model | Bayes | NA | NA | NA | NA | NA | 18 | 2 | 4 | 13 |
| Bo et al.(set2) | 2023 | CECT | Gray level normalization | Z-score normalization | Machine learning | Radiomic & clinical model | SVM | NA | NA | NA | NA | NA | 18 | 2 | 4 | 13 |
| Bo et al.(set3) | 2023 | CECT | Gray level normalization | Z-score normalization | Machine learning | Radiomic & clinical model | LR | NA | NA | NA | NA | NA | 20 | 5 | 2 | 10 |
| Bo et al.(set4) | 2023 | CECT | Gray level normalization | Z-score normalization | Machine learning | Radiomic & clinical model | RF | NA | NA | NA | NA | NA | 16 | 2 | 6 | 13 |
| Bo et al.(set5) | 2023 | CECT | Gray level normalization | Z-score normalization | Machine learning | Radiomic & clinical model | NN | NA | NA | NA | NA | NA | 22 | 5 | 0 | 10 |
| Bo et al.(set6) | 2023 | CECT | Gray level normalization | Z-score normalization | Machine learning | Radiomic & clinical model | LightGBM | NA | NA | NA | NA | NA | 18 | 5 | 4 | 10 |
| Bo et al.(set7) | 2023 | CECT | Gray level normalization | Z-score normalization | Machine learning | Radiomic & clinical model | XGBoost | NA | NA | NA | NA | NA | 20 | 3 | 2 | 12 |
| Qin et al. | 2021 | CECT | Filtering using 4 filters (Average, Disk, Gaussian, and Motion) to expand feature extraction | NA | Machine learning | Radiomic & clinical model | LASSO | Random split | 34 | 5 | 12 | 19 | 22 | 1 | 8 | 6 |
| Chen et al.(set1) | 2023 | CECT | NA | Z-score normalization | Machine learning | Radiomic & clinical model | NN | 10-fold cross validation | 26 | 8 | 5 | 56 | 11 | 3 | 2 | 25 |
| Chen et al.(set2) | 2023 | CECT | NA | Z-score normalization | Machine learning | Radiomic & clinical model | LR | 10-fold cross validation | 24 | 8 | 7 | 56 | 11 | 3 | 2 | 25 |
| Chen et al.(set3) | 2023 | CECT | NA | Z-score normalization | Machine learning | Radiomic & clinical model | RF | 10-fold cross validation | 26 | 8 | 5 | 56 | 13 | 4 | 0 | 24 |
| Chen et al.(set4) | 2023 | CECT | NA | Z-score normalization | Machine learning | Radiomic & clinical model | Bayes | 10-fold cross validation | 27 | 15 | 4 | 49 | 11 | 4 | 2 | 24 |
| Chen et al.(set5) | 2023 | CECT | NA | Z-score normalization | Machine learning | Radiomic & clinical model | SVM | 10-fold cross validation | 26 | 8 | 5 | 56 | 11 | 3 | 2 | 25 |
| Chen et al.(set6) | 2023 | CECT | NA | Z-score normalization | Machine learning | Radiomic & clinical model | XGBoost | 10-fold cross validation | 25 | 8 | 6 | 56 | 12 | 4 | 1 | 24 |
| Zhu et al. | 2021 | CECT | NA | NA | Machine learning | Radiomic & clinical model | LR | Random split | 9 | 2 | 2 | 20 | NA | NA | NA | NA |
| Chakraborty et al. | 2022 | CECT | Intensity filtering | Min-Max normalization | Machine learning | Radiomic & clinical model | AdaBoost | 3-fold cross validation | 32 | 11 | 7 | 89 | NA | NA | NA | NA |

TP true positive; TN true negative; FP false positive; FN false positive; NA not available; RF random forest; LR logistic regression; NN neural network; LASSO least absolute shrinkage and selection operator.
