# Supplementary figures and images for: Diagnostic Performance of Computed Tomography–Based Artificial Intelligence for Early Recurrence of Cholangiocarcinoma: Systematic Review and Meta-Analysis
Source: J Med Internet Res. 2025 Sep 18;27:e78306. doi: 10.2196/78306 (PMC12491900; doi:10.2196/78306)

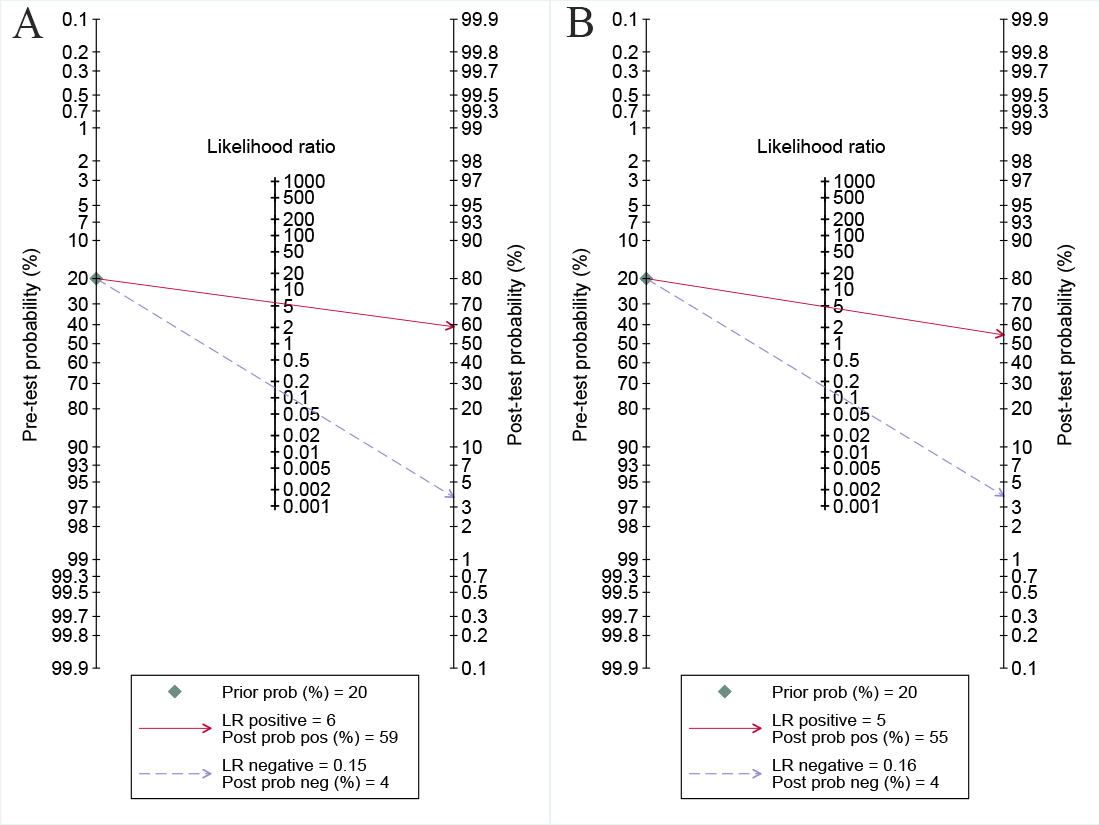

Supplement: Multimedia Appendix 6 [file jmir_v27i1e78306_app6.png]

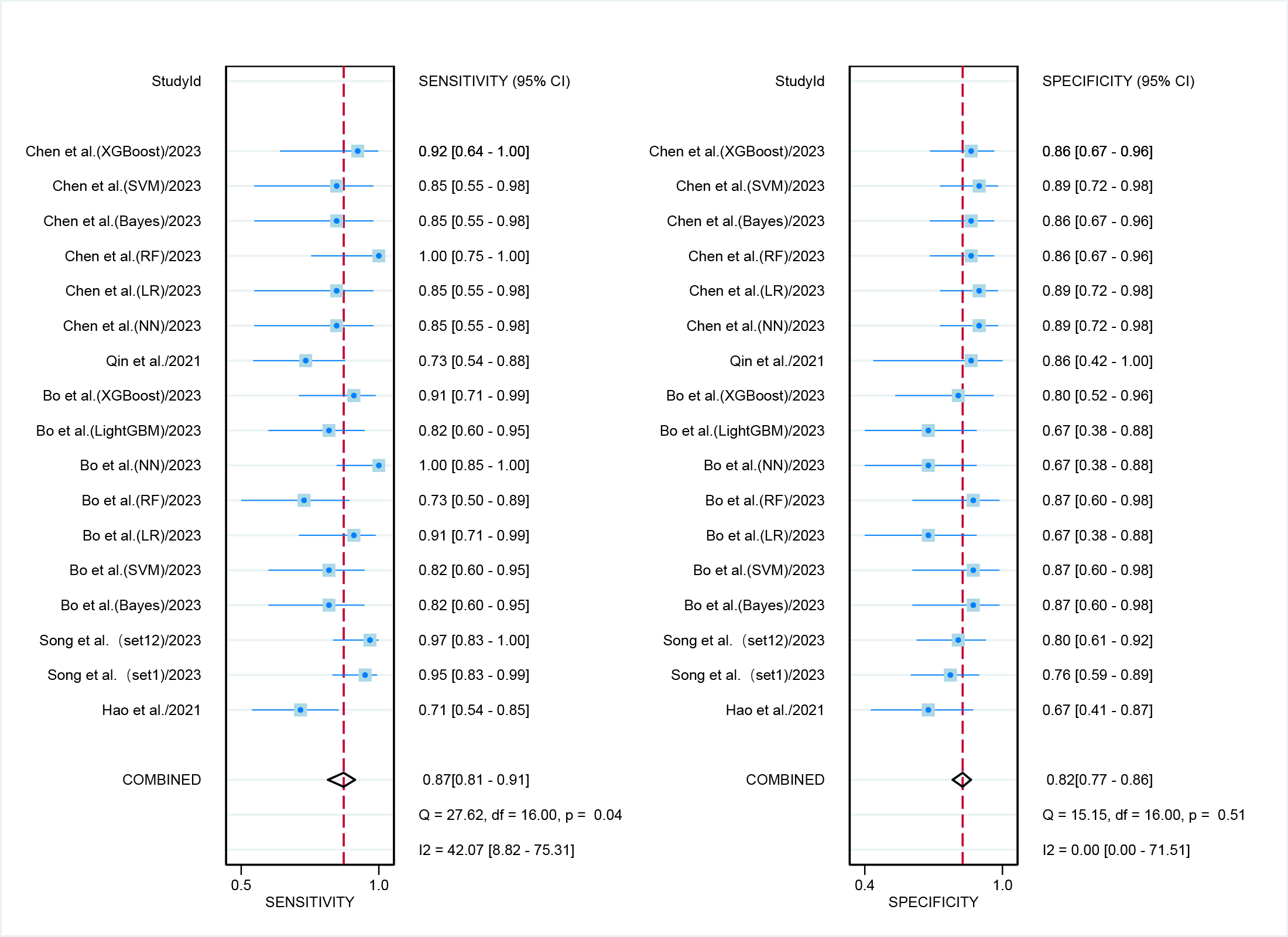

Supplement: Multimedia Appendix 7 [file jmir_v27i1e78306_app7.png]

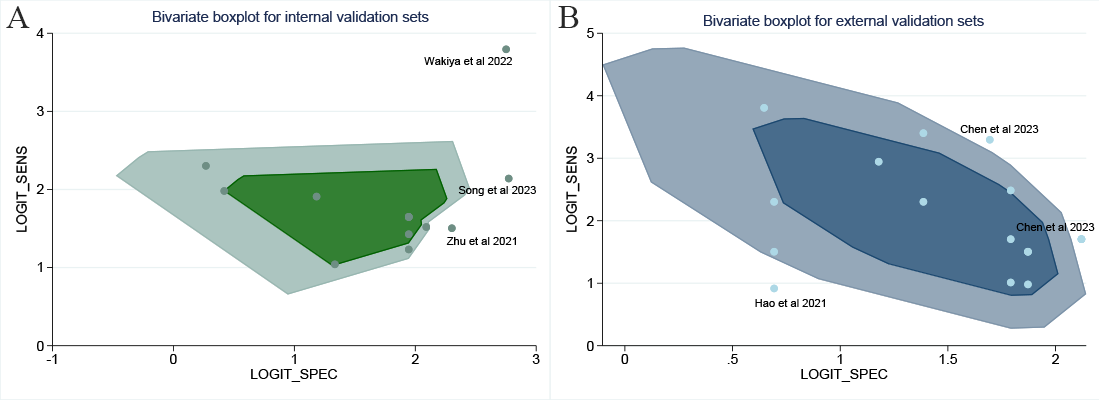

Supplement: Multimedia Appendix 10 [file jmir_v27i1e78306_app10.png]
