# Supplementary material for: Diagnostic Performance of Computed Tomography–Based Artificial Intelligence for Early Recurrence of Cholangiocarcinoma: Systematic Review and Meta-Analysis
Source: J Med Internet Res. 2025 Sep 18;27:e78306. doi: 10.2196/78306 (PMC12491900; doi:10.2196/78306)
Supplement: Multimedia Appendix 8 [file jmir_v27i1e78306_app8.docx]

**Table S1.** Subgroup analysis and meta-regression analysis of the diagnostic performance of CT-based artificial intelligence for early recurrence of cholangiocarcinoma within external validation sets.

| Subgroup | Studies, n | Sensitivity(95%CI) | Meta-regression *P*-value | Specificity(95%CI) | Meta-regression *P*-value |
| --- | --- | --- | --- | --- | --- |
| **Type of Cholangiocarcinoma** |  |  | 0.05 |  | 0.68 |
| IHC | 16 | 0.88 [0.83 - 0.92] |  | 0.82 [0.78 - 0.86] |  |
| PHC | 1 | 0.74 [0.50 - 0.98] |  | 0.86 [0.60 - 1.00] |  |
| **Reference standard** |  |  | 0.53 |  | <0.001 |
| Pathology and clinical imaging follow-up | 15 | 0.84 [0.79 - 0.89] |  | 0.83 [0.78 - 0.87] |  |
| Clinical imaging follow-up | 2 | 0.96 [0.91 - 1.00] |  | 0.78 [0.68 - 0.88] |  |
| **AI model** |  |  | 0.62 |  | 0.74 |
| Radiomic model | 1 | 0.72 [0.49 -0.95] |  | 0.67 [0.43 - 0.90] |  |
| Radiomic &clinical model | 16 | 0.88 [0.84 - 0.92] |  | 0.83 [0.79 - 0.87] |  |
| **Data splitting method** |  |  | 0.09 |  | 0.11 |
| Random split | 3 | 0.90 [0.82 - 0.99] |  | 0.78 [0.68 - 0.88] |  |
| K-fold cross validation | 7 | 0.86 [0.78 - 0.95] |  | 0.85 [0.80 - 0.91] |  |

IHC intrahepatic cholangiocarcinoma; PHC perihilar cholangiocarcinoma.
