# Supplementary material for: Diagnostic Performance of Computed Tomography–Based Artificial Intelligence for Early Recurrence of Cholangiocarcinoma: Systematic Review and Meta-Analysis
Source: J Med Internet Res. 2025 Sep 18;27:e78306. doi: 10.2196/78306 (PMC12491900; doi:10.2196/78306)
Supplement: Multimedia Appendix 9 [file jmir_v27i1e78306_app9.docx]

**Table S1.** Sensitivity analysis.

|  | Datasets, n | Sensitivity(95%CI) | Specificity(95%CI) | AUC(95%CI) | DOR(95%CI) |
| --- | --- | --- | --- | --- | --- |
| **Internal validation sets** |  |  |  |  |  |
| **Without outliers in Bivariate Box plot ^a^** | 10 | 0.83 [0.78 - 0.87] | 0.82 [0.75 - 0.88] | 0.87 [0.84 - 0.90] | 22.28[14.43 - 34.40] |
| **Without low-quality studies ^b^** | 7 | 0.90 [0.80 - 0.95] | 0.84 [0.71 - 0.91] | 0.93 [0.91 - 0.95] | 43.43 [12.31 - 153.31] |
| **External validation sets** |  |  |  |  |  |
| **Without outliers in Bivariate Box plot ^c^** | 12 | 0.88 [0.82 - 0.93] | 0.80 [0.74 - 0.85] | 0.87 [0.84 - 0.90] | 30.48 [17.27 - 53.79] |
| **Without low-quality studies ^b^** | 4 | 0.88 [0.70 - 0.96] | 0.76 [0.44 - 0.84] | 0.80 [0.77 - 0.84] | 21.79 [5.74 - 82.68] |

AUC area under the curve; DOR diagnostic odds ratio**.**

**^a^** Excluded Song et al.2023, DOI: 10.1007/s12072-023-10487-z, Wakiya et al. 2022, DOI: 10.1038/s41598-022-12604-8, Zhu et al.2021, DOI: 10.1038/s41598-021-97796-1

**^b^** Low quality means that the study was rated as having at least one high-risk item in the Revised QUADAS-2 tool.

^c^ Excluded Hao et al.2021, DOI: 10.1109/embc46164.2021.9630029, Chen et al. 2023, DOI: 10.1016/j.compbiomed.2023.107612
